# Teaching accelerated nursing students’ self‐care: A pilot project

**DOI:** 10.1002/nop2.384

**Published:** 2019-09-27

**Authors:** Cheryl Green

**Affiliations:** ^1^ Department of Nursing Southern Connecticut State University New Haven Connecticut

**Keywords:** nurse educators, nursing students, self‐care, stress reduction

## Abstract

**Aim:**

A benchmark of 4 has been determined for the reduction of self‐reported stress by nursing students’ status post 5 weeks of holistic educational activities and interventions provided by a nurse educator.

**Design:**

Provision 5 in the American Nurses Association Code of Ethics for Nurses with Interpretive Statements emphasizes the duty of the nurse to not only promote the health and safety of others, but to self as well (ANA, 2015, Code of ethics with interpretive statements, http://Nursebooks.org). A self‐care for nurses’ pilot project was trialled with 25 accelerated nursing students over the course of 5 weeks. Holistic education programmes were facilitated by a nurse educator uninvolved in providing clinical or classroom education to the students.

**Methods:**

The Standards for Quality Improvement Reporting Excellence (SQUIRE) guidelines are used in this pilot project as a framework to explore standardization of education of nursing students about self‐care in nursing programmes and to promote positive health behaviours and student nurses’ insight into how nurses’ self‐care can have an impact on patient outcomes. The self‐care pilot project introduced the importance of self‐care for the pre‐licensure nursing student by teaching healthy eating, physical exercise, the value of sleep, use of positive affirmations and aromatherapy to a cohort of accelerated nursing students over the course of 5 weeks. The Star Model of Knowledge Transformation was the theoretical framework for the pilot study. Two questionnaires were used by the principal investigator to obtain participant data, the Project Participant Questionnaire and the Final‐Year Group Questionnaire.

**Results:**

On completion of the self‐care for nurses’ pilot, the nursing students reported a reduction in stress and an increased ability to cope with stress after exposure to different holistic stress reduction strategies. An average benchmark of 4.36 was achieved indicating that the nursing students’ self‐care had improved status post the interactive teaching intervention.

Self‐care taught to pre‐licensure nursing students by nurse educators can enhance their self‐awareness of the importance of stress reduction and care of themselves while enduring the academic rigour and simultaneous clinical practicum experiences in nursing programmes.

Applying self‐care behaviours to reduction of stress for nursing students may be of benefit to of students as they transition from the pre‐licensure to graduate nurse roles. Hence, teaching health behaviours that are self‐protective and contribute to maintaining safe clinical environments for nurses and the patients in their care.

## 1 INTRODUCTION

Adult students that choose to enter accelerated nursing programmes enter academic study that is mentally and physically challenging. For example, courses that are four credits and completed in a 15‐week semester may be abbreviated to 5 or 7 weeks. Accelerated nursing students therefore must adapt study habits that include daily: hours of reading and reviewing of content to provide safe, quality care to patients and successfully prepare for classroom theory examinations.

Accelerated nursing programmes provide an opportunity for adult learners with bachelor and master's degrees in other fields, to complete a bachelor of science in a nursing programme within 11–18 months. According to the American Association of Colleges of Nursing (AACN), in 2016 in the United States, 47 states had accelerated programmes. Guam, the District of Columbia and the Virgin Islands, also have accelerated nursing programmes. Nationwide, there are approximately 272 accelerated baccalaureate programmes (AACN, 2017).

Baccalaureate accelerated nursing programmes maintain the rigour of 4‐year traditional nursing programmes. However, nursing students in accelerated programmes complete intensive courses and clinical practicums within a shortened time. Accelerated nursing students attend nursing programmes full‐time. Some accelerated nursing students may have children, spouses, significant others, own their homes, maintain full‐time and/or part‐time employment, or are caring for an ill parent or spouse. Given the rigour of the accelerated nursing programmes, students may choose to be unemployed while attending school and take out substantial loans to pay for their tuition. The latter can contribute to accelerated nursing students feeling stressed, have disturbance in sleep/rest patterns and neglect their own self‐care.

## 2 BACKGROUND

### 2.1 Stress

According to Cestari‐Feitosa, Barbosa, Florencio, de Paula Pessoa‐Mendes, and Moreira‐Magalhaes (2017), “Currently, the word stress is understood as an experience of tension, irritation, where an organism reacts to physical or psychological components when a situation that causes fear, excitement or confusion appears, which can trigger manifestations of depression, tachycardia, digestive disorder, among other things” (p. 191). The rigour and complexity of nursing education can prove challenging, as the accelerated nursing student completes courses in 3‐ to 7‐week intervals and begins a clinical experience in a healthcare setting providing care to patients. The stress of nursing school can at times, seem overwhelming.

### 2.2 Self‐care

Self‐care is defined by the World Health Organization (2014) as activities promoting health and preventing illness or disease that can involve the guidance of healthcare providers or be performed independently without a provider. Examples of self‐care activities are nutrition, sleep, complimentary alternative therapies, exercise and hygiene. Nurse educators teach nursing students to take care of patients in community and inpatient settings. However, minimal emphasis is given to teaching nursing students how to provide self‐care to themselves (Nevins & Sherman, 2016).

### 2.3 Factors having an Impact on Student Nurses Performance

#### 2.3.1 Sleep

Sleep deprivation in nursing students was examined by Thomas, McIntosh, Lamar, and Allen (2017) with a sample of 179 undergraduate pre‐licensure nursing students. The methodology applied was The Sleep Deprivation of Nursing Students, which is 4‐point Likert scale questionnaire that contained 45 questions that addressed motor vehicle safety, safety during work and clinical experiences, use of sleep‐inducing aids, personal sleep habits, caffeine stimulant use and spare time activities. Findings were that 155 students received <5 hr of sleep a day; however, needed seven or more hours a day to feel rested. All the 179 students participating in the study reported being sleep deprived.

Accelerated nursing students are required to complete 8 to 12‐hr clinical days for application of theories of adult, maternal–child and paediatric health. Because accelerated nursing students are second‐career students, some of these students are more likely to have dual responsibilities such as child‐rearing and part‐time employment. Sleep deprivation can become a problem. Being fully awake and alert to care for ill patients in the clinical environment and prepare for nursing school examinations may cause accelerated nursing students to use sleep aids to fall asleep and stimulants to maintain wakefulness (Thomas et al., 2017).

According to Thomas et al. (2017), 35 nursing students ingested stimulants to stay awake, 56 used sleep aids to fall asleep, 166 maintained employment during their academic and clinical studies of 8 hr or more a week, 158 had 12 hr clinical rotations, while 64 students both worked and had clinical rotations. While 144 students reported their 12‐hr clinical to be too long, more than half preferred the 12‐hr clinical and work experiences because it permitted additional time to study on another day of the academic week. Of the 179 nursing students, 174 believed they provided safe practice at clinical and 174 reported being able to perform their employment‐related responsibilities.

Students had awake periods of 17 to 19 hr. However, the students had no awareness of how sleep deprivation could affect their health. Findings indicated that nursing students needed education on the impact of sleep deprivation on the potential development of chronic and acute health issues. Thomas et al. (2017) noted: “Nurse educators and nurse managers must collaborate to reduce the number of consecutive clinical and work shifts, limit overtime and better educate students on the negative impact of sleep deprivation” (p. 87).

#### 2.3.2 Nutrition and exercise

Nursing students’ intake stimulants, such as caffeine, chocolate or sweets, to stay awake and alert during the academic and clinical day(s). This behaviour is not without insight by the nursing students. Interestingly, Chow and Kalischuk (2008) noted in an exploratory study that nursing students tend to engage in self‐care activities. However, it is the rigour of nursing academics, assignments and clinical that contribute to nursing students reported lack of self‐care activities; sleep, exercise, intake of healthy foods and adequate fluid hydration (Nevins & Sherman, 2016).

#### 2.3.3 Complementary alternative therapies

Examples of complementary alternative therapies (CAT) include aromatherapy, reflective journaling, foot reflexology, positive affirmations and breathing for sleep. Chan and Schaffrath (2017) applied participatory action enquiry using baccalaureate nursing students and use of integrative healthcare modalities in a nursing core curriculum. In their study that lasted over a period of two semesters, two cohorts (cohort 1‐26/60; cohort 2‐20/60) participated. Twenty‐six nursing students volunteered to participate in the first cohort that originally consisted of 60 nursing students. Twenty nursing students participated in the second cohort.

The second cohort had two nursing faculty who were holistic mentors for the students. The concept of applied participatory action enquiry was used by the nursing faculty mentors with the nursing student volunteers in the providing of CAT to patients in the clinical setting. According to Chan and Schaffrath, of the 219 patients who were approached by nursing students and nurse faculty mentors to participate in the study, 147 (67%) agreed to have one or more CAT modalities (i.e. foot reflexology, lavender aromatherapy and breathing for sleep) performed. Results of this study indicated that identified core concepts consisting of holistic care introduced by nurse educators into the curriculum via patient care practice situations with nursing students could validate relational pedagogy. Hence, the integration of health promotion, into patient care provided by pre‐licensure nursing students, of a holistic nature, is beneficial to nursing students in learning the value of CAT, not only for the patients in their care, but for themselves as well.

#### 2.3.4 Health behaviours

Blake, Stanulewicz and Griffith (2017) provided a questionnaire to preregistered nurses addressing healthy lifestyle behaviours and health promotion. The questionnaire was completed by 493 preregistered nurses and the measures covered body satisfaction and health promotion. Study results found that 89.5% of preregistered nurses led unhealthy lifestyles and exhibited low self‐esteem with regard to dissatisfaction with their body. Ironically, the preregistered nurses believed that nurses should be role models for health. Self‐perception and self‐esteem could be correlated to the adaptation of self‐confidence, assertiveness, care of the physical body with exercise and healthy eating (Blake et al., 2017; Spurgas, 2005).

According to Iacobucci, Daly, Linedell, and Griffin (2013), a healthy sense of self can be not only beneficial to the nurse and their professional values but can have an impact on the delivery of patient care. Thus, teaching nursing students the importance of self‐care and working in healthy environments can be beneficial as students begin to transition to the role of pre‐licensure nursing student to graduate nurse. Teaching student nurses self‐care in accordance with the American Nurses Association's Code of Ethics for Nurses with Interpretive Statements, Provision 5, can help student nurses view the importance of creating ethical workplace environments that are safe (and healthy) for nurses and their colleagues (ANA, 2015).

Self‐care studies with nursing students at the baccalaureate level (Nevins & Sherman, 2016) note that educational support addressing self‐care interventions of exercise, diet, increasing knowledge about complementary alternative therapies (CAT), general health, hydration and reduction of stress, can be of benefit. Nursing students in accelerated nursing programmes can experience a significant amount of stress given the intensity of academic rigour of accelerated programmes and the brevity of completion of course work averaging 11–18 months. Sleep deprivation, poor eating habits, negative self‐concepts with regard to health perceptions and lack of exercise can have an impact on health. Negative thoughts compounded by stress associated with fear of not successfully meeting course work and clinical expectations may impair accelerated nursing students’ ability to manage their stressors.

Carlson and Warne (2007) found that when nurses working in healthcare environments are healthy, there is evidence that the self‐care of the nurses has a positive impact on the health promotion provided to patient populations served by the nurses. Therefore, health promotion behaviours (Bryer, Cherkis, & Raman, 2013) of pre‐licensure nursing students may be affected when nurse educators teach students about self‐care techniques. The latter can directly correlate with improving nursing students’ self‐concept (Horneffer, 2006) with regard to improving self‐care.

## 3 METHODS

### 3.1 Ethical approval

Institutional Review Board (IRB) approval was obtained in 2016. Inclusion criteria were that participants had to be accelerated nursing students that attended Southern Connecticut State University (SCSU) during the 2016–2017 academic year. Human subject ethical approval was also obtained. The principal investigator reviewed with potential participants, the risks and benefits of study participation. Participants, after review of the consent form, signed the form and were provided a copy of the document. Confidentiality of all study questionnaires was maintained through use of random numbers selected by participants. At the end of the pilot project, participants were provided a $25.00 gift card to Trader Joe's, a grocery chain that specializes in healthy foods.

### 3.2 Standards for quality improvement reporting excellence

The Standards for Quality Improvement Reporting Excellence (SQUIRE, see McCallum, 2009) are guidelines that serve as a framework for quality improvement initiatives in health care. For this pilot study, the standardization of the education of nursing students about self‐care in nursing programmes to promote positive health behaviours and student nurses’ insight as to how self‐care can have an impact on patient outcomes was explored.

### 3.3 Theoretical framework

The Star Model of Transformation is an evidence‐based practice model that provides a theoretical framework for five concepts underlying how knowledge is integrated into practice. These five concepts are configured within a five‐pointed star as various forms of knowledge, in a relational sequential pattern. The five major stages of knowledge transformation include the following: (a) Discovery Research; (b) Evidence Summary; (c) Translation to Guidelines; (d) Practice Integration; and (e) Process Outcome Evaluation (Stevens, 2012, 2013). The knowledge integration involved in this pilot study was the education of nursing students about self‐care while in nursing school.

### 3.4 Demographics

Twenty‐five accelerated nursing students, out of a class of 32 students, volunteered to participate. The participants attended an accelerated 12‐month nursing programme at SCSU, a public university in New England. The purpose of the study was to examine the benefits of self‐care education provided to accelerated nursing students. Demographics (Table 1) included the following: ages of the accelerated nursing students 18–24 (*N* = 5), 25–34 (*N* = 17), 35–44 and (*N* = 3) and sex, 80.0% (*N* = 20) female and 20.0% (*N* = 5) male. Marital status of participants was as follows: 20.0% (*N* = 5), married; 72.0% (*N* = 18) single; 4.0% (*N* = 1) separated; and 4.0% (*N *− 1) divorced. With regard to ethnicity, 92.0% (*N* = 23) were Caucasian, 4.0% (*N* = 1) African American and 4.0% (*N* = 1) Other. Educational backgrounds of the accelerated nursing students included 88.0% (*N* = 22) with a bachelor's degree and 12.0% (*N* = 3) with a master's degree.

**Table 1:** Below are the frequencies for each the demographic variables

Valid	Frequency	Percent	Valid Percent	Cumulative Percent
Age				
18–24	5	20.0	20.0	20.0
25–34	17	68.0	68.0	88.0
35–44	3	12.0	12.0	100.0
Total	25	100.0	100.0	
Ethnicity				
Caucasian	23	92.0	92.0	92.0
African American	1	4.0	4.0	96.0
Other	1	4.0	4.0	100.0
Total	25	100.0	100.0	
Highest degree				
Bachelor's	22	88.0	88.0	88.0
Master's	3	12.0	12.0	100.0
Total	25	100.0	100.0	
Marital status				
Married	5	20.0	20.0	20.0
Single	18	72.0	72.0	92.0
Separated	1	4.0	4.0	96.0
Divorced	1	4.0	4.0	100.0
Total	25	100.0	100.0	
Gender				
Female	20	80.0	80.0	80.0
Male	5	20.0	20.0	100.0
Total	25	100.0	100.0	

For this self‐care pilot project for pre‐licensure nursing students, data were obtained from 25 accelerated nursing students. Data enquiries were collected on students’ self‐report of ways they engage in stress reduction behaviours and strategies (i.e. having a pet, taking a warm bath and gardening). The purpose of this qualitative study was to examine the benefits of self‐care for nursing students in an accelerated nursing programme through teaching the students about holistic ways to provide themselves self‐care.

### 3.5 Self‐care

At the beginning of the study, the accelerated nursing students were provided education on the American Nurses Association's Code of Ethics for Nurses with Interpretive Statements, Provision 5. Provision 5 states, “The nurse owes the same duties to self as to others, including the responsibility to promote health and safety, preserve wholeness of character and integrity, maintain competence and continue personal and professional growth” (American Nurses Association, 2015, V). The accelerated nursing students were provided, over a period of 5‐weeks, teaching on the importance of self‐care in helping themselves in their ability to cope with stress associated with their academic and clinical experiences in nursing school. Additionally, interactive activities were provided for students to reinforce information provided to them on sleep benefits, healthy eating, aromatherapy, positive affirmations and exercise.

The accelerated nursing students were asked to identify at least five stress reduction techniques they have used while in nursing school, that they have found effective (Table 2). Different methods of stress reduction were identified on the Project Participant Questionnaire (PPQ). The PPQ was developed by the principal investigator and Dr. Charles Morgan, a psychiatrist at the Bridgeport Hospital in Bridgeport, Connecticut. The PPQ also inquired about anxiety, stress and distraction associated with the clinical skill of medication administration to assist nursing students in understandings the clinical impact of stress and lack of self‐care on patient outcomes. Nursing students circled the method(s) they used to reduce their stress (Tables 3 and 4). The following eight methods of stress reduction were identified: physical exercise 32.0% (*N* = 8); playing with a pet 4.0% (*N* = 1); yoga (*N* = 1); music 4.0% (*N* = 1); gardening/nature 4.0% (*N* = 1); physical exercise with music 32.0% (*N* = 8); prayer 8.0% (*N* = 2), 4.0% (*N* = 1); taking a bath 8.0% (*N* = 2). The top four stress reduction techniques identified by the nursing students were physical exercise, physical exercise with music, prayer and taking a bath.

**Table 2:** Pre‐test questions

		Frequency	Percent	Valid Percent	Cumulative Percent
Pre: Ever feel anxious when administering medications on the clinical unit					
Valid	No	6	24.0	24.0	24.0
	Yes	19	76.0	76.0	100.0
	Total	25	100.0	100.0	
Pre: Ever had a near miss or an actual medication error					
Valid	No	8	32.0	33.3	33.3
	Near miss	13	52.0	54.2	87.5
	Actual medication error	1	4.0	4.2	91.7
	Both a and b	2	8.0	8.3	100.0
	Total	24	96.0	100.0	
Missing	System	1	4.0		
Total		25	100.0		
Pre: Find administering medications to patients a stressful experience on clinical unit					
Valid	No	11	44.0	44.0	44.0
	Yes	14	56.0	56.0	100.0
	Total	25	100.0	100.0	
Pre: Ever been distracted on clinical unit when administering medications to patients					
Valid	No	13	52.0	54.2	54.2
	Yes	11	44.0	45.8	100.0
	Total	24	96.0	100.0	
Missing	System	1	4.0		
Total		25	100.0		
Pre: Method(s) you use to reduce your stress and anxiety					
Valid	Meditation and Physical Ex	2	8.0	8.0	8.0
	Meditation, Physical exerc	2	8.0	8.0	16.0
	Music and Taking a bath	1	4.0	4.0	20.0
	No answer	1	4.0	4.0	24.0
	Physical exercise and Gard	1	4.0	4.0	28.0
	Physical exercise and Music	1	4.0	4.0	32.0
	Physical Exercise and Music	1	4.0	4.0	36.0
	Physical exercise, Gardeni	1	4.0	4.0	40.0
	Physical exercise, Music	4	16.0	16.0	56.0
	Physical exercise, Prayer	3	12.0	12.0	68.0
	Prayer	1	4.0	4.0	72.0
	Prayer, Music, Taking a b	1	4.0	4.0	76.0
	Prayer, Music, Taking a ba	1	4.0	4.0	80.0
	Prayer, Yoga, and Taking a	2	8.0	8.0	88.0
	Yoga and Physical exercise	1	4.0	4.0	92.0
	Yoga and Playing with a Pe	1	4.0	4.0	96.0
	Yoga, Playing with my kids	1	4.0	4.0	100.0
	Total	25	100.0	100.0	

**Table 3:** Post‐test Questions

		Frequency	Percent	Valid Percent	Cumulative Percent
Post: Ever feel anxious when administering medications on the clinical unit					
Valid	No	7	28.0	28.0	28.0
	Yes	18	72.0	72.0	100.0
	Total	25	100.0	100.0	
Post: Ever had a near miss or an actual medication error					
Valid	No	8	32.0	34.8	34.8
	Near miss	15	60.0	65.2	100.0
	Total	23	92.0	100.0	
Missing	System	2	8.0		
Total		25	100.0		
Post: Find administering medications to patients a stressful experience on clinical unit					
Valid	No	11	44.0	44.0	44.0
	Yes	14	56.0	56.0	100.0
	Total	25	100.0	100.0	
Post: Ever been distracted on clinical unit when administering medications to patients					
Valid	No	9	36.0	36.0	36.0
	Yes	16	64.0	64.0	100.0
	Total	25	100.0	100.0	
Post: Method(s) you use to reduce your stress and anxiety					
Valid	Meditation, Physical exerc	8	32.0	32.0	32.0
	Music, Yoga, and Playing w	1	4.0	4.0	36.0
	Physical exercise	1	4.0	4.0	40.0
	Physical exercise and Music	1	4.0	4.0	44.0
	Physical exercise, Gardeni	1	4.0	4.0	48.0
	Physical exercise, Music,	8	32.0	32.0	80.0
	Physical exercise, Prayer	2	8.0	8.0	88.0
	Prayer and Music	1	4.0	4.0	92.0
	Prayer, Music, Gardening/*N*	2	8.0	8.0	100.0
	Total	25	100.0	100.0	

**Table 4:** Cross‐tabulation of Pre‐ and Post‐test

Question 1				
Pre: Ever feel anxious when administering medications on the clinical unit * Post: Ever feel anxious when administering medications on the clinical unit Cross‐tabulation				
Count		Post: Ever feel anxious when administering medications on the clinical unit		Total
		No	Yes	
Pre: Ever feel anxious when administering medications on the clinical unit	No	4	2	6
	Yes	3	16	19
Total		7	18	25
The exact sig. (*p* = 1.00) means there is no difference in the change in response from pre to post				

Chi‐square tests		
	Value	Exact sig. (two‐sided)
McNemar Test		1.000^a^
*N* of Valid cases	25	

Question 2				
Pre: Ever had a near miss or an actual medication error * Post: Ever had a near miss or an actual medication error Cross‐tabulation				
Count		Post: Ever had a near miss or an actual medication error		Total
		No	Near miss	
Pre: Ever had a near miss or an actual medication error	No	3	5	8
	Near miss	3	8	11
	Actual medication error	0	1	1
	Both a and b	1	1	2
Total		7	15	22

Could not calculate because not a square (PxP) matrix. Post only has No and Near miss as responses			
Chi‐square tests			
	Value	*df*	Asymptotic significance (two‐sided)
McNemar–Bowker Test	.	.	.^b^
*N* of Valid cases	22		

Question 3				
Pre: Find administering medications to patients a stressful experience on clinical unit * Post: Find administering medications to patients a stressful experience on clinical unit Cross‐tabulation				
Count		Post: Find administering medications to patients a stressful experience on clinical unit		Total
		No	Yes	
Pre: Find administering medications to patients a stressful experience on clinical unit	No	6	5	11
	Yes	5	9	14
Total		11	14	25

Chi‐square tests		
	Value	Exact sig. (two‐sided)
McNemar Test		1.000^a^
*N* of Valid cases	25	

Question 4				
Pre: Ever been distracted on clinical unit when administering medications to patients * Post: Ever been distracted on clinical unit when administering medications to patients Cross‐tabulation				
Count				
		Post: Ever been distracted on clinical unit when administering medications to patients		Total
		No	Yes	
Pre: Ever been distracted on clinical unit when administering medications to patients	No	4	9	13
	Yes	5	6	11
Total		9	15	24

Chi‐square tests		
	Value	Exact sig. (two‐sided)
McNemar Test		0.424^a^
*N* of Valid Cases	24	

a Binomial distribution used.

b Computed only for a PxP table, where P must be >1.

Self‐care education was provided by the principal investigator. The self‐care interventions taught to the nursing students included: healthy eating, exercise, the benefits of sleep, aromatherapy and positive affirmations. Self‐care activities and educational content were taught before and after an Adult Health class during the summer of 2017. Nursing students voluntarily approached a table that was set up approximately 20 feet from their classroom in the hallway. For each of the 5 weeks of the pilot study, students voluntarily approached the table and obtained reading materials on the topic of the week. Also provided on the table were activities that enhanced learning through interaction. The following educational interviews were provided: Week 1 Benefits of Sleep, Week 2 Healthy Eating, Week 3 Exercise, Week 4 Aromatherapy and Week 5 Positive Affirmations.

### 3.6 Final‐Year Student Group Questionnaire

The Final‐Year Student Group Questionnaire was a 3‐item questionnaire administered at the end of the study. This questionnaire was developed by the principal investigator. The Final‐Year Student Group Questionnaire asked the accelerated nursing students to provide feedback about educational materials and information provided over the course of the 5‐week self‐care educational intervention and whether these resources were beneficial.

### 3.7 Holistic self‐care interventions

#### 3.7.1 Week 1

Nursing students were provided education on the benefits of sleep. Fact sheets were provided to the students with recommendations on how to train their body to sleep by going to bed at the same time each night. The avoidance of stimulants, caffeine and nicotine at least 4–6 hr before bedtime and maintaining a cool dark environment. Classical music was played at a low volume in the background. Students were encouraged to obtain sleep/rest that would not be disrupted. Nursing students were also encouraged not to bring their textbooks to bed, but use the bed for only sleeping or intimacy. A raffle was held and students won either new pillows or an instrumental music CD for relaxation and promotion of sleep/rest.

#### 3.7.2 Week 2

Healthy eating education was discussed with the nursing students and handouts provided on quick healthy meal preparation within 30 min or less. No student allergies were reported. Fresh fruits and vegetables were provided for students to snack on during their breaks from lecture. Fruits and vegetables included apples, oranges, carrots, grapes, bananas, broccoli and sliced green peppers. Additionally, gluten‐free, multigrain crackers and bottled spring water were provided.

#### 3.7.3 Week 3

Nursing students learned about exercise. Handouts were provided on exercise safety, the importance of stretching before and after exercise and hydration (and protein intake). The benefits of aerobic, flexibility and strength and resistance training were taught. Motivational exercise music was quietly played in the background. Students were encouraged to take walks around the university's campus during breaks between their classes or play a game of ball or Frisbee with peers.

Exercise was introduced to the nursing students via the use of exercise stations that included five pound weights, jump ropes and a stationary cycle. Fact sheets on the benefits of maintaining cardiovascular health, strengthening exercises (i.e. weight lifting) and using yoga and Pilates for stretching and balance were provided. A raffle was held, and students won jump ropes, weights and exercise bands for strengthening and stretching.

#### 3.7.4 Week 4

Students were provided information on different natural aromatherapy oils and the oils’ therapeutic use and indications (i.e. lavender for relaxation and bergamot for stress reduction). No student allergies were reported. Placed on the table were small pieces of multicoloured felt so that a small amount of the oil(s) could be placed on the felt piece for students to smell. An aromatherapy fact sheet was provided to the nursing students that gave an overview of essential oils and how they are used. Samples of orange citrus, peppermint, bergamot and lavender oils were provided to students on small swatches of the felt on their request. Bergamot oil was presented as an essential oil that can be used for stress reduction. Lavender was discussed as being used to promote relaxation. Interestingly, during this education session, all 25 of the nursing students participating in the pilot project reported that the use of the Bergamot oil as they listened to their Adult Health lecture (medical–surgical nursing class), helped them feel relaxed and improved their ability to concentrate.

#### 3.7.5 Week 5

Positive affirmations are self‐affirming statements that enhance one's belief in themselves and their abilities to accomplish an identified goal or task. Students were provided positive affirmations with positive themes focusing on test and quiz examination success, provision of patient‐focused care exhibiting nursing caritas and the achievement of a registered nurse licensure on successful completion of the nursing programme. Students were all provided a book that provided tips on resume development and interview skills to prepare them for employment as graduate nurses. The book also contained some positive affirmations.

## 4 RESULTS

The Final‐Year Group Questionnaire (Table 5) provided an opportunity for participants to anonymously report whether they experienced any benefit(s) from the stress reduction techniques taught to them over the course of 5 weeks. These stress reduction techniques included aromatherapy, healthy eating, exercise, the benefits of sleep and positive affirmations. The benchmark of 4 was determined on a Likert scale of 1 (Not at all)–5 (Very much so). The response frequencies for the Final‐Year Group Questionnaire yielded the following results for three questions: For Question 1, *do you plan to utilize some of the stress and anxiety techniques you learned?* 8 (32.0%) nursing students selected 4 and 15 (60.0%) selected 5. Question 2, *did you find the educational materials about stress and anxiety helpful?* 13 (52.0%) students selected 4 and 12 (48.0%) selected 5. Lastly, for Question 3*, do you believe you can use stress, anxiety and distraction reduction techniques while administering medications?* 12 (48.0%) selected 4 and 9 (36.0%) selected 5.

**Table 5:** The response frequencies for the three final‐year group questions

		Frequency	Percent	Valid Percent	Cumulative Percent
Do you plan to utilize some of the stress and anxiety techniques you learned					
Valid	2	1	4.0	4.0	4.0
	3	1	4.0	4.0	8.0
	4	8	32.0	32.0	40.0
	Very much so	15	60.0	60.0	100.0
	Total	25	100.0	100.0	
Did you find the educational materials about stress and anxiety helpful					
Valid	4	13	52.0	52.0	52.0
	Very much so	12	48.0	48.0	100.0
	Total	25	100.0	100.0	
Do you believe you can use stress, anxiety and distraction reduction techniques while administering medications					
Valid	Not at all	1	4.0	4.0	4.0
	3	3	12.0	12.0	16.0
	4	12	48.0	48.0	64.0
	Very much so	9	36.0	36.0	100.0
	Total	25	100.0	100.0	

	Descriptive statistics				
	*N*	Minimum	Maximum	Mean	*SD*
Do you plan to utilize some of the stress and anxiety techniques you learned	25	2	5	4.48	0.770
Did you find the educational materials about stress and anxiety helpful	25	4	5	4.48	0.510
Do you believe you can use stress, anxiety and distraction reduction techniques while administering medications	25	1	5	4.12	0.927

On review of the descriptive statistics for the Final‐Year Questionnaire, frequencies for the 25 accelerated nursing student participants were as follows: Question 1, minimum of 2, maximum of 5, a mean of 4.48 and a standard deviation of 0.770; Question 2, minimum of 4, maximum of 5, mean of 4.48 and a standard deviation of 0.510. And Question 3, minimum of 1, maximum of 5, mean of 4.12 and a standard deviation of 0.927. These results suggest that the interventions of the educational techniques of aromatherapy, exercise, healthy eating, the benefits of sleep and positive affirmations were beneficial to the nursing students in their reduction of stress, anxiety and distraction. The latter, potentially influencing students’ responses positively, in the management of stress. The average for the benchmark was 4.36 indicating that students’ self‐care had improved status post the interactive teaching intervention.

During this study, 1 of the 25 students experienced an actual medication error. The student reported during the study, experiencing anxiety, stress and distraction while administering medications to patients and having a prior near miss medication error that was intercepted by the student and her clinical instructor. However, by week 4 of the study, the student reported an increase awareness of stress and identified ways to cope and decrease stress prior to providing patient care on the clinical unit (i.e. sleep and exercise).

## 5 DISCUSSION

The findings of this qualitative study suggest that nursing students experience stress while on the clinical unit. However, with the intervention of teaching nursing students self‐care health behaviours, students’ may have an increased awareness of their need to reduce their own stress. An American College Health Association (ACHA) survey in 2014 noted that 43.7% of students reported experiencing *more than average stress* during college and 47.4% shared that their academics, within the last 12 months, were *traumatic or difficult to handle* (ACHA, 2014, pp. 14–15).

## 6 CONCLUSION

Persistent stress experienced by nursing students can impair their performance and affect their health and well‐being (Drew et al., 2016). Accelerated nursing students in particular, because of the rapid pace of their academic programmes, may benefit from learning self‐care while in nursing programmes. However, all nursing students would benefit from self‐care education despite the length of their academic programmes.

## 7 RELEVANCE TO CLINICAL PRACTICE

As nurse educators, it is imperative that we mentor and teach the next generation about the importance of self‐care. The American Nurses Association's Code of Ethics for Nurses with Interpretive Statements, Provision 5, which emphasizes the duty of the nurse to not only promote the health and safety of others, but to self as well (ANA, 2015), should be a guiding factor in the implementation and emphasis of self‐care in nursing programmes. The latter will create healthier pre‐licensure nurses that have a keen awareness of how their failure to care for themselves can potentially have an impact on patient outcomes and their own health.

## CONFLICT OF INTEREST

The author has contributed solely to this manuscript and has no identified conflicts of interests.
