# Supplementary material for: Teaching accelerated nursing students’ self‐care: A pilot project
Source: Nurs Open. 2019 Sep 27;7(1):225–34. doi: 10.1002/nop2.384 (PMC6917926; doi:10.1002/nop2.384)
Supplement: Supplementary file 1 [file NOP2-7-225-s001.docx]

Standards for Quality Improvement Reporting Excellence

Checklist

SQUIRE: The purpose of the SQUIRE is to provide a framework for the reporting of new knowledge in healthcare with the goal of improving health care safety and quality.

**Title and Abstract**

1. Title

This manuscript entitled, Teaching Accelerated Nursing Students Self-Care: A Pilot Project, involves an initiative to improve the health behaviors of prelicensure nursing students while they are undergoing academic studies and clinical practice in nursing school. Thereby teaching them to maintain positive health behaviors as they transition to the graduate nurse and registered nurse roles. The health and well-being of nurses also equates to quality and safe patient care delivery. (Page 1)

1. Abstract

Under the Methods section of the Abstract, the Star Model of Knowledge Transformation is identified as the theoretical framework used in the manuscript. Two questionnaires used within the pilot study are the Project Participant Questionnaire and the Final-Year Group Questionnaire. (Page 1)

1. Methods

Under the Methods section the Star Model of Knowledge Transformation is identified as the theoretical framework used in the manuscript. Two questionnaires used within the pilot study are the Project Participant Questionnaire developed by the principal investigator and a colleague, Dr. Charles Morgan and the Final-Year Group Questionnaire developed by the principal investigator. (Pages 8-11)

**Reference**

Standards for Quality Improvement Reporting Excellence (2017). *SQUIRE: Promoting* *Excellence in Healthcare Improvement Reporting*. Retrieved from <http://www.squire->

statement.org/
